# Experimental X-ray and DFT Structural Analyses of M_12_L_8_ Poly-[*n*]-catenanes Using exo-Tridentate Ligands

**DOI:** 10.1021/acs.inorgchem.2c01290

**Published:** 2022-06-30

**Authors:** Javier Martí-Rujas, Sijie Ma, Antonino Famulari

**Affiliations:** † Dipartimento di Chimica Materiali e Ingegneria Chimica “Giulio Natta”, Politecnico di Milano, Via Luigi Mancinelli 7, Milan 20131, Italy; ‡ Center for Nano Science and Technology@Polimi, Istituto Italiano di Tecnologia, Via Pascoli 70/3, Milan 20133, Italy; § INSTM Consorzio Interuniversitario Nazionale per la Scienza e Tecnologia dei Materiali, Florence 50121, Italy

## Abstract

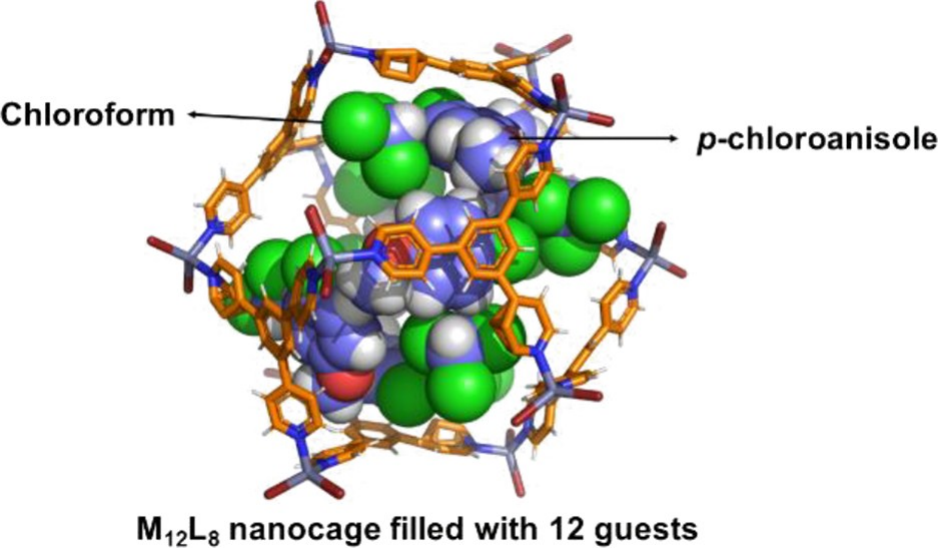

Despite their potential applications in host–guest chemistry, there are only five reported structures of poly-[*n*]-catenanes self-assembled by elusive **M_12_L_8_** icosahedral nanocages. This small number of structures of **M_12_L_8_** poly-[*n*]-catenanes is because self-assembly of large metal–organic cages (MOCs) with large windows allowing catenation by means of *mechanical bonds* is very challenging. Structural reports of **M_12_L_8_** poly-[*n*]-catenanes are needed to increase our knowledge about the self-assembly and genesis of such materials. Poly-[*n*]-catenane (**1·*p*-CT**) self-assembly of interlocked **M_12_L_8_** icosahedral cages (**M** = Zn(II) and L = 2,4,6-tris-(4-pyridyl)benzene (**TPB**)) including a new aromatic guest (*p*-chlorotoluene (***p*-CT**)) is reported by single-crystal XRD. Despite the huge internal **M_12_L_8_** voids (> 2500 Å^3^), *p*-CT is ordered, allowing a clear visualization of the relative host–guest positions. DFT calculations have been used to compute the electrostatic potential of the **TPB** ligand, and various aromatic guests (i.e., *o*-dichlorobenzene (***o*-DCB**), *p*-chloroanisole (***p*-CA**), and nitrobenzene (**NBz**)) included (ordered) within the **M_12_L_8_** cages were determined by single-crystal XRD. The computed maps of electrostatic potential (**MEPs**) allow for the rationalization of the guest’s inclusion seen in the 3D X-ray structures. Although more crystallographic X-ray structures and DFT analysis are needed to gain insights of guest inclusion in the large voids of **M_12_L_8_** poly-[*n*]-catenanes, the reported combined experimental/DFT structural analyses approach can be exploited to use isostructural **M_12_L_8_** poly-[*n*]-catenanes as hosts for molecular separation and could find applications in the *crystalline sponge* method developed by Fujita and co-workers. We also demonstrate, exploiting the *instant synthesis* method, in solution (i.e., ***o*-DCB**), and in the solid-state by *neat grinding* (i.e., without solvent), that the isostructural **M_12_L_8_** poly-[*n*]-catenane self-assembled with 2,4,6-tris-(4-pyridyl)pyridine (**TPP**) ligand and ZnX_2_ (where X = Cl, Br, and I) can be *kinetically* synthesized as crystalline (yields ≈ 60%) and amorphous phases (yields ≈ 70%) in short time and large quantities. Despite the change in the aromatic nature at the center of the rigid exo-tridentate pyridine-based ligand (**TPP** vs **TPB**), the *kinetic control* gives the poly-[*n*]-catenanes selectively. The dynamic behavior of the **TPP** amorphous phases upon the uptake of aromatic guest molecules can be used in molecular separation applications like benzene derivatives.

## Abstract 2

A combined X-ray and DFT structural analysis focusing on the host-guest chemistry of **M_12_L_8_** polycatenanes using exo-tridentate ligands is reported.

## Introduction

The self-assembly of discrete metal organic cages (**MOCs**) with well-defined voids is attracting much attention.^1^ Besides the exceptional symmetric structures,^2^ this finds functional applications in areas such as molecular separation,^1−5^ catalysis,^6−8^ and emergent behavior because of their internal nanoconfined space.^6,9−11^ One strategy to combine the structural properties of **MOCs** and metal–organic frameworks (**MOFs**)^12−18^ is by the preparation of poly-[*n*]-catenanes by mechanically interlocking metal organic cages^19−21^ through *mechanical bonds*.^22−27^ However, the synthesis of polycatenanes made of **MOCs** is not trivial because the cages need to have large windows where catenation can take place.^28−31^ The self-assembly of Platonic icosahedral **MOCs** is elusive with very few examples reported so far.^32,33^ Even more rare are the so-called one-dimensional (1D) **M_12_L_8_** poly-[*n*]-catenanes which are formed by the interlocking of **M_12_L_8_** nanocages in one crystallographic direction. This is because enthalpic and entropic aspects play a crucial role in the self-assembly of such large host guest systems.^4,28−31^ Using exo-tridentate 2,4,6-tris-(4-pyridyl)pyridine (**TPP**)^34,35^ or 2,4,6-tris-(4-pyridyl)benzene (**TPB**)^36^ ligands and ZnX_2_ (where X = Cl and I), a new class of poly-[*n*]-catenanes self-assembled with large **M_12_L_8_** icosahedral nanocages have been synthesized in solution. The π–π interactions arising from the aromatic central part of the ligands and the presence of aromatic templating solvents are crucial in the formation of the crystalline interlocked **M_12_L_8_** nanocages.

Achieving control over the products obtained in the synthesis of **M_12_L_8_** poly-[*n*]-catenanes is very important. The crystallization method can give rise to different products. For instance, slow against fast crystallization might result in thermodynamic or kinetic structures, respectively. A powerful approach to control the products is by using very fast crystallization (i.e., instant synthesis)^36^ which minimizes the error-checking process, so the *kinetic* phase is obtained homogeneously and in good quantities.^39−43^ The synthesis in the absence of an aromatic templating solvent forms amorphous poly-[*n*]-catenane,^37^ which also shows dynamic behavior in the presence of various guest molecules. Thus, in the solution state, the formation of crystalline **M_12_L_8_** poly-[*n*]-catenanes is a guest-driven process. X-ray structures including aromatic guest molecules in **M_12_L_8_** poly-catenated icosahedral nanocages are very limited, with only four structures reported describing precise host–guest interactions.^35−38^ Therefore, more X-ray crystallographic data where the structures can give a broad structural view and can be used to carry out DFT calculations are needed to increase our knowledge about **M_12_L_8_** poly-[*n*]-catenanes and their guest behavior.

**Scheme 1:**
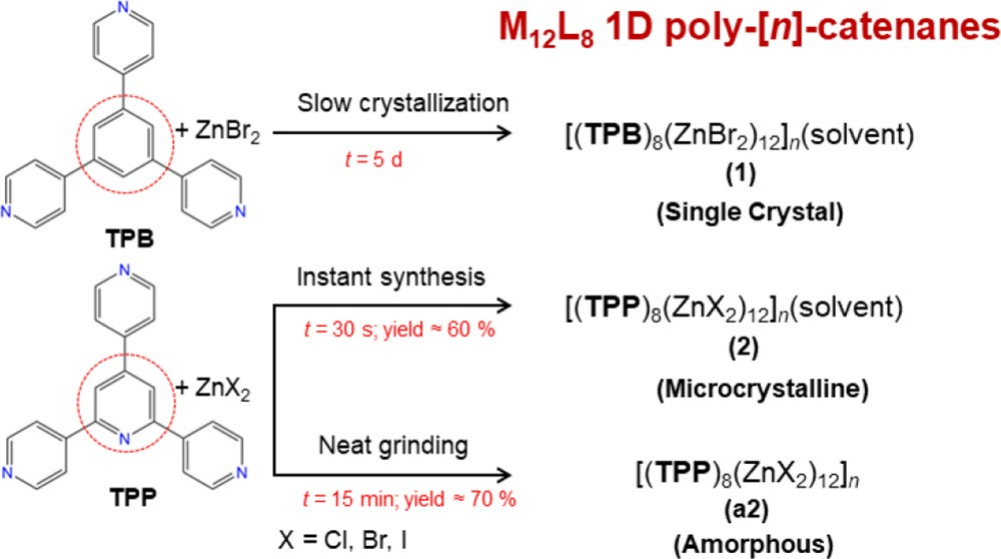
Crystallization Methods Used To Synthesize the Poly-[*n*]-catenanes Described in This Work Using exo-Tridentate Ligands **TPB** and **TPP** and Zinc Halides The central ring in **TPB** and **TPP** is highlighted with a red-dashed circle.

Here, we report a combined experimental X-ray and theoretical DFT (density functional theory) structural analysis of **M_12_L_8_** poly-[*n*]-catenane crystals self-assembled with **TPB** and ZnBr_2_ in the presence of templating aromatic solvents (Scheme 1). DFT calculations have been carried out to determine the maps of electrostatic potential (**MEPs**) of the host **TPB** and guest molecules *p*-chlorotoluene (***p*-CT**), *o*-dichlorobenzene (***o*-DCB**), *p*-chloroanisole (***p*-CA**), and nitrobenzene (**NBz**) reported by SC-XRD to better understand the host–guest affinity. This is useful in guest inclusion reactions which can be important to exploit the **M_12_L_8_** poly-[*n*]-catenanes in the selective separation of molecules. The effect of the ligand core (i.e., the central ring), in terms of π–π interactions, has been studied by synthesizing isostructural **M_12_L_8_** poly-[*n*]-catenanes self-assembled with **TPP** and ZnX_2_ (where X = Cl, Br, and I) under *kinetic control* (Scheme 1). As demonstrated by powder XRD data, this is the first report of poly-[*n*]-catenanes using **TPP** and ZnBr_2_. The kinetic control given by the instant synthesis (yields ≈ 60%) allows the selective crystallization of the polycatenane product, excluding other structures that might be favored under slower crystallization conditions (i.e., thermodynamic products).^34,36^ Moreover, a series of amorphous poly-[*n*]-catenanes (**a2**) have also been prepared in the solid state by neat grinding, leading to noncrystalline solids (yields ≈ 70%) that can uptake different aromatic guests from liquid phases. The dynamic behavior shown by the isostructural **M_12_L_8_** poly-[*n*]-catenanes self-assembled with **TPB** or **TPP** and ZnX_2_ (where X = Cl, Br, I) opens up many applications in areas such as molecular separation, gas adsorption, or drug delivery.

## Results and Discussion

### Single-Crystal X-ray Structure of **1·*p*-CT**

**Figure 1:**
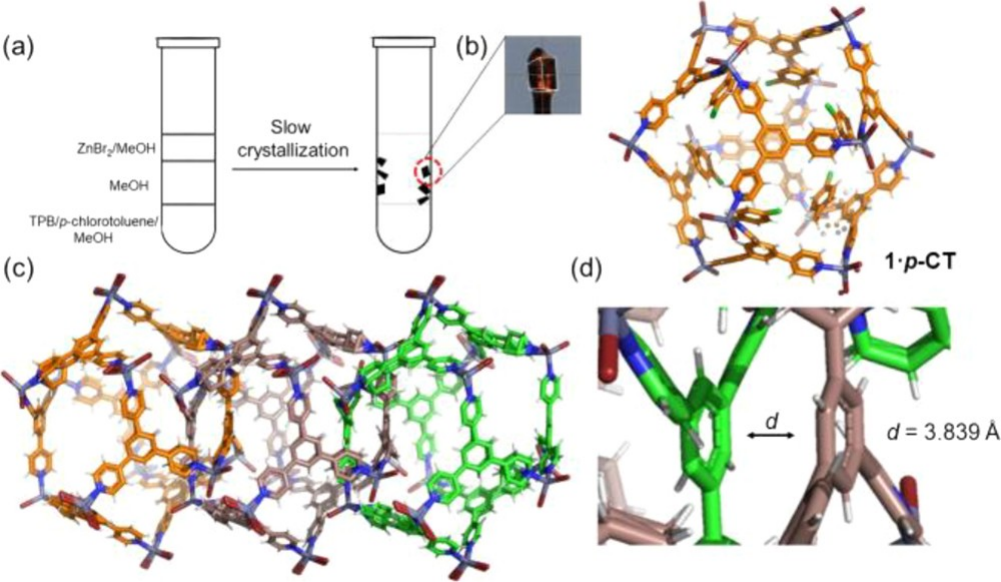
(a) Cartoon showing the slow crystallization experiment giving large single crystals of **1·*p-*CT**. (b) Single-crystal X-ray structure of **1·*p*-CT** showing one **M_12_L_8_** nanocage and six ***p*-CT** guest molecules viewed approximately along the *c*-axis. (c) View of three **M_12_L_8_** nanocages linked by the mechanical bond. (d) Zoomed view showing the aromatic–aromatic (benzene–benzene) distance among **TPB** ligands in the interlocked **M_12_L_8_** cages.

A single crystal of the **TPB**-ZnBr_2_**M_12_L_8_** poly-[*n*]-catenane was obtained using a layering **TPB** solution of ***p*-CT** and a methanolic solution of ZnBr_2_ (Figures 1a and S1). The crystal structure was solved in the trigonal system (*R-*3) with the lattice parameters (100 K): *a* = *b* = 37.9460(6) Å, *c* = 15.7786(3) Å, α = β = 90°, γ = 120°; *V* = 19675.7(6) Å^3^ with *Z* = 3. The formula of the complex from the X-ray data is [(ZnBr_2_)_12_(**TPB**)_8_]_*n*_·4(C_7_H_7_Cl) (**1·*p*-CT**). In the asymmetric unit, there is one ligand and one-third of a second ligand **TPB** and ***p*-CT** with an occupancy factor of 0.6663 for the ordered guest, according to the single-crystal XRD data.

The Zn(II) metal centers located at the vertices of the **M_12_L_8_** icosahedron display a tetrahedral geometry with three Zn–N (2.036, 2.037, and 2.030 Å) coordination bonds and four Zn–Br bonds ranging from 2.363 to 2.333 Å. In **1·*p*-CT**, the **M_12_L_8_** icosahedrons are defined as “*opened icosahedrons*” (Figure 1b) because there are eight triangular faces that do not contain **TPB** giving rise to large windows (13.5 × 21.4 Å). These large openings in the **M_12_L_8_** nanocages are important for allowing the material to explore the best ligand–ligand interactions during the self-assembling process and the *mechanical bond* formation.^22−27,31^ Two adjacent triangular empty faces allow the interlacing of other **M_12_L_8_** nanocages. Like in other catenanes, efficient aromatic–aromatic interactions are crucial for a good π–π stacking interaction stabilizing the catenane’s structure.^19−21,31^ In the present case, the **TPB** benzene–benzene distance is 3.839 Å (Figure 1c).

In **1·*p*-CT**, the **M_12_L_8_** “*framework*” is slightly more disordered than in the chloride isostructural version.^36^ One of the two ZnBr_2_ units and one pyridine ring are disordered over two positions (Figure S6). This is important because the pyridine mobility has been used to explain, in combination with DFT calculations, the dynamic behavior of poly-[*n*]-catenane regarding *crystal-to-crystal* guest release and inclusion from and into the **M_12_L_8_** nanocages.^38^ The nanocages are doubly interlocked and expand along the [001] crystallographic direction (Figure 1c). Removing in silico the guest molecules from the **M_12_L_8_** nanocages, the *free* volume obtained is 6656.52 Å^3^ (i.e., 33.8% of unit cell volume; Figure S4). Importantly, the 100 K structure of **1·*p*-CT** does not have continuous channels but isolated **M_12_L_8_** nanocages. The isolated voids are important because they reduce the mobility of the guest content, decreasing the “entropic penalty” due to the disordered solvent. If the voids were connected, the flow of the solvent will make the structure less stable, in particular, at room-temperature conditions.

### Host–Guest Interactions in Isostructural **M_12_L_8_** Poly-[*n*]-catenanes

**Figure 2:**
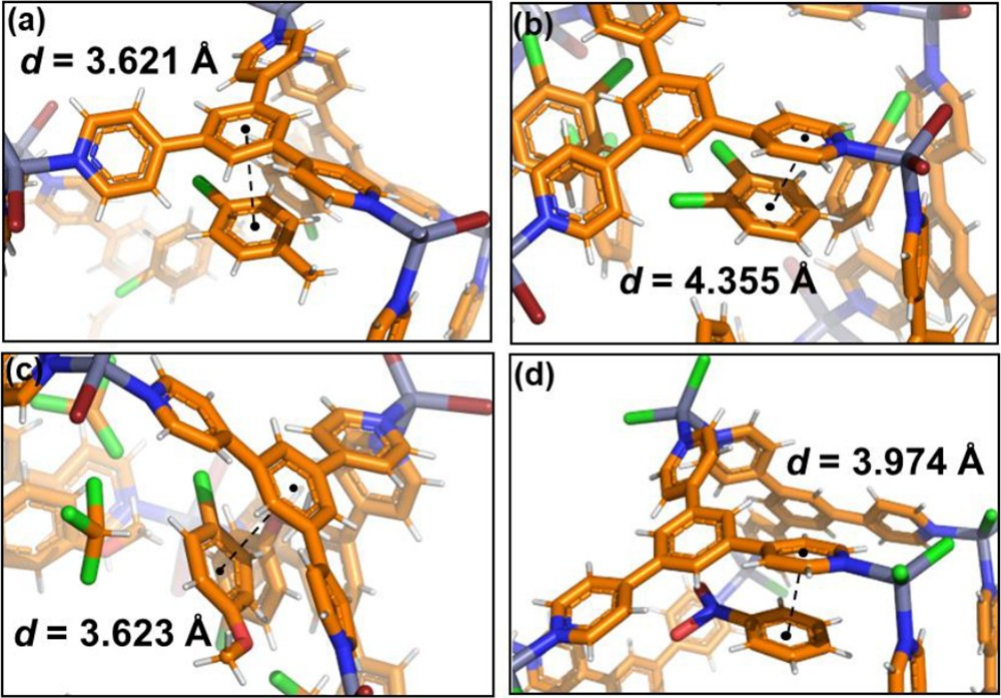
Augmented view of the SC-XRD structures of **1·*p*-CT** (a), **1·*o*-DCB** (b), **1·*p*-CA** (c), and 1*·NBz(36) (d). The star mark (*) indicates that the terminal halide in Zn(II) is Cl. The distance among the host (**TPB**) and the guests is considering the centroids of the rings.

While the guest inclusion in small voids is widely reported, the encapsulation and 3D structural characterization of guests within very large cavities (∼2500 Å^3^) are much more challenging due to the lack of efficient guest interactions with the host walls.^26^ The X-ray crystal structures show that the longer guest molecules, ***p*-CT** (Figure 2a) and ***p*-CA** (Figure 2c),^44^ are oriented in such a way that the aromatic interactions are among benzene–benzene rings with distances ca. 3.621 and 3.623 Å. However, the shorter guest, ***o*-DCB** (Figure 2b), interacts via *benzene*–*pyridine* interactions, although the *benzene*–*benzene* interaction is also possible. Interestingly, in this case, the host–guest distance is longer (*d* = 4.355 Å). Figure 2d also depicts the **TPB**-ZnCl_2_ poly-[*n*]-catenane including nitrobenzene (1*·NBz)^36^ in which also the aromatic–aromatic interaction occurs between the pyridine of **TPB** and the benzene ring of the nitrobenzene. In this case, the host–guest distance is ∼4 Å.

The different distances among hosts and guests interacting via aromatic–aromatic interactions shown in Figure 2 have been compared with models that used more accurate DFT approaches.^45^ Hobza and co-workers computed the interaction energies, considering *benzene*–*benzene*, *benzene*–*pyridine,* and *pyridine*–*pyridine* dispersion interactions (π–π). From their work, it is observed that the stronger interactions are in the pyridine–benzene dimers, whereas the benzene–benzene dispersion interactions are weaker. The observed experimental X-ray structural data in Figure 2 show that the shortest host–guest distances are in the *benzene*–*benzene* interactions and not in *pyridine*–*benzene,* as explained above. In our opinion this can be due to the disorder in the pyridine rings in the **M_12_L_8_** framework.

### DFT Calculations

What is determining the disposition of the ordered guest molecules “*glued*” to the **TPB** ligand? Clearly, more poly-[*n*]-catenane structures including aromatic guest molecules are necessary to increase our knowledge about the guest inclusion in **TPB M_12_L_8_** poly-[*n*]-catenanes. Although it cannot be considered as the only factor controlling the disposition of the guests in the **M_12_L_8_** cages, as geometry and size are important, the electrostatic potential of the **TPB** ligand, and of each guest molecule, has an influence on the stabilization of the ordered guests and hence the formation of the poly-[*n*]-catenane in its crystalline form. The presence of a nitrogen instead of a carbon atom in the benzene ring (i.e., the core of the **TPB** ligand) reduces its polarizability, creates a dipole, and decreases the spatial extent of the electron density.^45^ With the aim to better understand the host–guest affinity in **TPB M_12_L_8_** poly-[*n*]-catenanes, DFT calculations have been carried out on the **TPB** ligand and guest molecules discussed (***p*-CT**, ***o*-DCB**, ***p*-CA**, and **NBz**).

**Figure 3:**
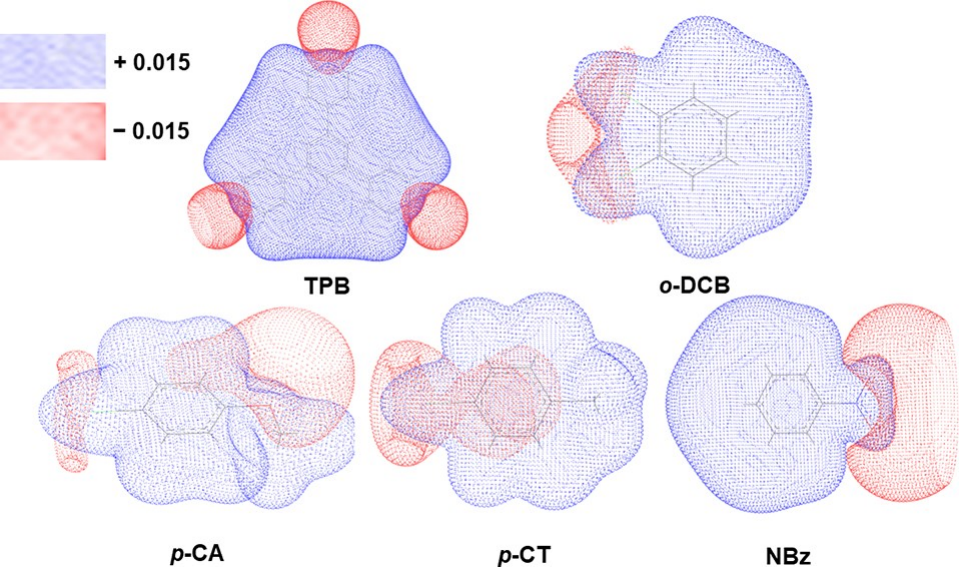
DFT electrostatic potential calculated for the ligand **TPB** and the four guest molecules ***p*-CT**, ***o*-DCB**, ***p*-CA**, and **NBz** discussed in the text. The electropositive and electronegative regions are represented in blue and red, respectively.

Figure 3 shows the **MEPs** calculated at the PBE/DNP level of approximation (roughly comparable to PBE/6-31G**, see Supporting Information), which have been employed in several recent studies.^46−53^ The dotted blue and red areas represent the positive and negative electrostatic potential regions, respectively (i.e., the more electropositive and electronegative areas). The differences in the host–guest interactions can also be rationalized by the different electrostatic potential surfaces of guest molecules with respect to that of the host ligand (i.e., the size and shape of guests are also the aspects influencing the host–guest interactions). In fact, the central region of **TPB** is positive and thus preferentially interacts with the negative surfaces of guest molecules. It is worth to note that, in the poly-[*n*]-catenane system (i.e., the DFT is calculated on the **TPB** ligand only), the coordination with metals increases this effect on the corresponding MEP. Both ***p*-CT** and ***p*-CA** have their positive areas (the methyl group) oriented toward the pyridine region (i.e., the negative region of **TPB**). In addition, we observe the specific orientation of guest CHCl_3_ molecules (see Figure 2c) in the crystalline architecture, with the “activated” C–H (partially positive) group pointing toward the bromide bonded to Zn metal (distance lower than the sum of van der Waals radii), while Cl atoms orient to the positive MEP regions of ***p*-CA**.

### M_12_L_8_ Icosahedral Cage’s Dynamic Behavior upon Guest Identity

Depending on the guest molecules, the lattice parameters of the isostructural poly-[*n*]-catenanes show significant variations that are worth to be analyzed. Table 1 displays a summary of structural parameters discussed in the text, including the host–guest distances and the benzene–benzene interactions constituting the mechanical bond among other relevant distances. One aspect shown in Table 1 worth to mention is that the **M_12_L_8_** cage height oscillates from 11.940 to 13.373 Å. This is because while the lattice parameters along the mechanical bond (*c*-axis) vary from 15.779 to 16.718 Å (Δ 0.939 Å), the interlocking π–π distances in all the materials remain quite stable (from 3.657 to 3.839 Å (Δ 0.182 Å)).

**Table 1:** List of Structural Parameters in the Described Structures **1·*p*-CT**, **1·*o*-DCB, 1·*p*-CA**, and 1*·NBz

	1·*p-*CT	1·*o-*DCB	1·*p*-CA	1*·NBz
cell parameter *a*, *b* (Å)	37.946	37.937	38.080	37.380
cell parameter *c* (Å)	15.779	16.718	15.957	16.097
cell volume (Å^3^)	19,676	20,837	20,039	19,479
**M_12_L_8_** interlocked cage height (Å)^a^	11.940	13.061	12.181	13.373
**M_12_L_8_** noninterlocked cage height (Å)^b^	19.618	20.322	20.712	20.548
host–guest distance (Å)^c^	3.621	4.355	3.623	3.974
interlocking π–π (Å)^d^	3.839	3.657	3.776	3.742

a Distance among the centroids of the benzene rings in the **TPB** ligands in a double-interlocked **M_12_L_8_** cage viewed along the *c*-axis.

b Distance from the top to the bottom in a single (noninterlocked) **M_12_L_8_** cage, taking the centroid of the central benzene ring of **TPB**.

c Distance calculated, taking the centroid of the **TPB** aromatic ring and the centroid of the aromatic guest.

d Distance among the two closest centroids in the **TPB** benzene rings forming the mechanical bond.

This indicates that within the 1D rod of interlocked **M_12_L_8_** cages, the dynamic behavior is mainly along the mechanical bond (*c*-axis). If we consider only the structures self-assembled with **TPB** and ZnBr_2_, we observe that the lattice parameters *a* and *b* are very similar despite hosting different guests: type and quantities of guests. Additionally, the single-crystal XRD data of a **TPB**-ZnBr_2_ polycatenane, including toluene measured at room temperature (**1·Tol**), show a *c*-axis with the lattice parameter equal to 15.531 Å, which is so far the shortest observed in this type of catenanes (Figure S7).^54^ This aspect is important as it shows that there is a significant dynamic behavior of **M_12_L_8_** poly-[*n*]-catenanes along the mechanical bond direction when it is compared to *a* and *b* lattice parameters (Figure S9).

Therefore, as observed from the X-ray data, the main **M_12_L_8_** icosahedral distortion is along the *c*-axis which is the propagating direction of the 1D chains of **M_12_L_8_** cages. This also has an important role regarding the relative movement of the 1D rods, which has been demonstrated by DFT that such movement (along the *c*-axis) does not imply a significant energy cost considering the potential energy surfaces of the poly-[*n*]-catenane chains.^38^ Additionally, we should consider that the *c*-axis reflects the projection of the metal–ligand bond. We can observe that the variations of the tilt angle of the ligand with respect to the *c*-axis do not significantly perturb the metal–ligand bond lengths. Thus, we can vary these tilt angles by changing the *c*-axis while maintaining the complex bond lengths almost unchanged. Furthermore, the intercage van der Waals forces can remain unchanged, elongating or shortening the *c*-axis.

### Isostructural **M_12_L_8_** Poly-[*n*]-catenanes as Potential Crystalline Sponges

Understanding *a priori* which guest molecules can be included in the **M_12_L_8_** nanocages forming the poly-[*n*]-catenanes can be of much interest, for instance, to use this class of materials in the *crystalline sponge method*(55−58) recently developed by Fujita and co-workers. There is not a unique MOF material that can be used in a general way as a crystalline sponge, but depending on the nature of the guest molecule (i.e., size, geometry, polarity, etc.) that has to be “*crystallized*,” the researcher needs to find the best metal organic material to be used.^58^ The fact that poly-[*n*]-catenanes can uptake guest molecules via *crystal*-*to*-*crystal* reactions,^38^ and that can be synthesized using **TPB** with various zinc halides (isostructural),^59^ gives the opportunity to the user to choose the most suitable MOF (i.e., the chloride, bromide, or the iodide version, considering the scattering power of the halogen).^60^ This has an important role regarding absorption effects but also the ease of crystallization. In this regard, DFT calculations can be very useful to determine the electrostatic potential of the guest molecule which can provide valuable information regarding guest inclusion in the **M_12_L_8_** nanocages. Such aspects are crucial to exploit all the potential of the crystalline sponge method.^58,60^

### Instant Synthesis of Poly-[*n*]-catenanes Using 2,4,6-Tris-(4-pyridyl)pyridine with ZnX_2_ (where X = Cl, Br, and I)

Because the self-assembling process is very sensitive to the host–guest interactions, and also due to the core ligand–ligand interactions (i.e., **TPB** in this case), we were interested to see if the poly-[*n*]-catenane can be obtained using the instant synthesis method with **TPP** ligand and zinc halides. The presence of a N instead of a C atom in the central ring of **TPP** decreases the spatial distribution of the electron density compared to that of **TPB**, which can have a direct effect on the self-assembly using the instant synthesis method. Even though it has been demonstrated that **TPP** forms single crystals of the poly-[*n*]-catenane with ZnCl_2_^34^ and ZnI_2_,^35^ but until now not with ZnBr_2_, it is not guaranteed that by using the instant synthesis method the product will be the **M_12_L_8_** poly-[*n*]-catenane. We found that the role played by the core of the **TPP** ligand in the instant synthesis is worth to be investigated.

**Figure 4:**
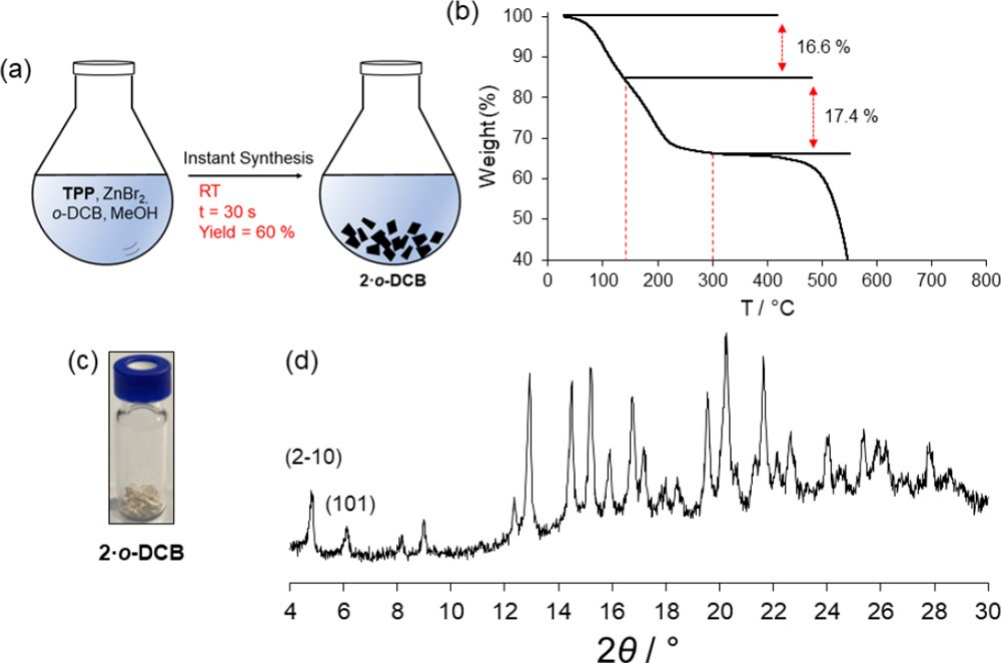
(a) Instant synthesis of **2·*o*-DCB** using **TPP** and ZnBr_2_ in *o*-DCB/MeOH. (b) TG plot of **2·*o*-DCB** showing the weight loss of the guest molecules. (c) Actual solid obtained after filtering. (d) Powder XRD pattern of the sample shown in (c).

The instant synthesis experiment was carried out using **TPP** and ZnBr_2_ (see Supporting Information). After filtration, the white powder (**2·*o*-DCB**) was left to equilibrate with the atmosphere for 5 days and then analyzed by powder XRD (Figure 4). The obtained product was 55 mg (yield 60%). The diffractogram clearly demonstrates that the sample is crystalline and isostructural to the solid prepared using **TPB,** as both powder XRD patterns show a good match (see Figure S13). It is important to highlight the reflections (2–10) and (101) which correspond to the peaks cutting the aromatic–aromatic **TPP** interactions in the poly-[*n*]-catenane. Thermogravimetric (TG) analysis has shown that the weight loss corresponding to the guest is 34%, which corresponds to 2.3 guest molecules per asymmetric unit (Figure 4). Another salient feature of **2·*o*-DCB** is that after being in contact with the atmosphere for 5 days, it still contains many entrapped solvents. This is because the **M_12_L_8_** nanocages are not connected, and to release the solvent, it is necessary to provide thermal energy. The instant synthesis has also been proved successful for the ZnCl_2_ and ZnI_2_^59^ isostructural poly-[*n*]-catenanes (Figure S11 and S12).

### Solid-State Synthesis of Poly-[*n*]-catenane Using 2,4,6-Tris-(4-pyridyl)pyridine with ZnX_2_ (where X = Cl, Br, and I)

**Figure 5:**
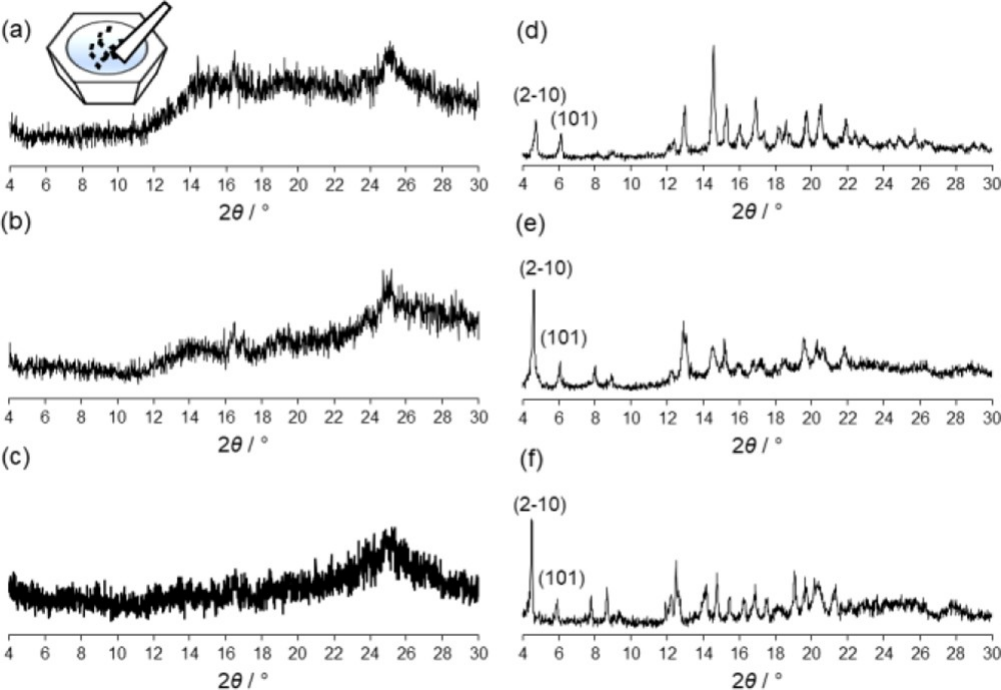
Powder XRD pattern showing the amorphous phases (**a2**) obtained after neat grinding of **TPP** and ZnCl_2_ (a), ZnBr_2_ (b), and (c) ZnI_2_ using a mortar and a pestle. Diffractograms corresponding to the crystalline phases (d–f) obtained after immersing the amorphous phases (a–c) in toluene/methanol overnight. The powder XRD corresponds to crystalline poly-[*n*]-catenanes after solvent uptake.

After showing that the **TPP**-ZnBr_2_ poly-[*n*]-catenane can be obtained by instant synthesis, our interest moved to its solid-state synthesis. The ability to synthesize in the solid state, solvent-free, a mechanically interlocked supramolecular structure is really notable, with only one reported case in the literature using **TPB** and ZnBr_2_.^37^ Moreover, in this case, it is worth to see the effect of a change in the core of the ligand (i.e., pyridine vs benzene) which might influence the electrostatic interactions and the formation of the mechanical bond in the solid state. Grinding **TPP** and ZnBr_2_ without solvent for 15 min using a mortar and a pestle results in an amorphous phase (**a2**). As demonstrated by powder XRD, the diffractogram shows the characteristic broad bumps (Figure 5a), which are indicative of no *long-range order* and the absence of the starting **TPP** ligand. The calculated yield is 72% (see Supporting Information).

To know if **a2** contains the mechanically interlocked **M_12_L_8_** structure, the solid was immersed in a mixture of toluene/methanol (6 mL:1 mL) and left stirring overnight under ambient conditions. After filtering the sample, the powder XRD pattern clearly shows that the amorphous phase turned crystalline (Figure 5d). The powder XRD pattern is similar to the one obtained by instant synthesis, with the two (2–10) and (101) peaks at low angles corresponding to the poly-[*n*]-catenane structure. This indicates that the mechanical bond is formed also in the amorphous phase **a2** by neat grinding. After the reconstruction induced by the templating guest effect, the aromatic stacking is very important, giving a *d*-spacing consistent with all the isostructural catenanes so far observed.

The solvent-free mechanochemical synthesis has also been extended to **TPP** and ZnCl_2_ or ZnI_2_ with yields ∼70%. In both cases, after grinding using a mortar and a pestle, the solid product also formed amorphous phases (Figure 5b,c). It is important to note that the amorphous phases are different from each other, as seen in Figure 5, but when immersed and stirred in toluene/methanol overnight, the amorphous phase adsorbs the solvent to give the crystalline poly-[*n*]-catenane (Figure 5e,f).^61^

Thus, after the reconstruction of the amorphous phase, it is confirmed that isostructural poly-[*n*]-catenanes can be obtained from amorphous phases by trapping different aromatic guest molecules. Clearly, the new amorphous poly-[*n*]-catenanes show an important dynamic behavior via an *amorphous-to-crystalline* transformation, which can be exploited in molecular separation applications using the amorphous poly-[*n*]-catenanes synthesized with **TPP** and zinc halides without a solvent. Importantly, a new library of mechanically interlocked materials can be added to those obtained with **TPB** and ZnX_2_.^36,37^

### Role of TPB and TPP Ligands and Zinc Halides in the Synthesis of M_12_L_8_ Poly-[*n*]-catenanes

It has been demonstrated that using **TPB** and **TPP** with ZnCl_2_, ZnBr_2_, and ZnI_2_, it is possible to prepare isostructural **M_12_L_8_** poly-[*n*]-catenanes via instant synthesis in their crystalline form in the presence of suitable aromatic guest molecules. The change in the central aromatic nature of the exo-tridentate ligand (i.e., benzene (**TPB**) or pyridine (**TPP**)) does not affect the final product if fast crystallization is used. This indicates that the selectivity achieved by the instant synthesis (kinetic control) is quite significant, as slow crystallization yields different structures. For instance, it has been observed that layering diffusion crystallization of **TPB** and ZnCl_2_ can also give 1D coordination polymers together with the poly-[*n*]-catenane.^36^ Likewise, **TPB** and ZnI_2_, by layering diffusion, yield a coordination polymer (i.e., not isostructural to that of **TPB** and ZnCl_2_) mixed with the polycatenane.^59^ Dehnen and co-workers reported a 1D coordination polymer using **TPP** and ZnCl_2_ under solvothermal conditions, and by layer crystallization, a similar, but not isostructural coordination polymer, is formed using ZnI_2_ and **TPP**.^34^ Interestingly, the poly-[*n*]-catenane self-assembled with **TPP** and ZnBr_2_ has not yet been reported by slow crystallization methods (i.e., under thermodynamic control). Therefore, in the solution state, if slow crystallization is used, the change of the **TPB** or **TPP** ligand and/or zinc halides really changes the outcome of the products, while using instant synthesis, a control of the products is achieved yielding six isostructural poly-[*n*]-catenanes.

Regarding the solid-state synthesis, we also have observed that for all the cases, an amorphous phase of the poly-[*n*]-catenane is formed selectively, as no other crystalline structures are formed. Because the products are not crystalline, we do not use the term isostructural amorphous phases, although when immersed in aromatic solvents, the poly-[*n*]-catenane is obtained upon guest uptake and reorganization via *amorphous-to*-*crystalline* transformation. Thus, using **TPB** or **TPP** ligands with ZnX_2_ (where X = Cl, Br, or I) does not affect the formation of the poly-[*n*]-catenane, whether in solution or in the solid state, if fast self-assembling methods are used under *kinetic control*.

## Conclusions

In conclusion, a **M_12_L_8_** poly-[*n*]-catenane self-assembled with **TPB**, ZnBr_2_, and the aromatic ***p*-CT** guest molecule has been reported using SC-XRD. The guest molecule has been resolved unambiguously by X-ray crystallography, allowing the precise observation of host–guest interactions, which is crucial to gain fundamental structural knowledge on **M_12_L_8_** poly-[*n*]-catenanes. DFT calculations have been carried out to calculate the maps of electrostatic potential of the ligand **TPB** and various aromatic guest molecules, yielding structural insights on the aromatic–aromatic host–guest interactions. The combined X-ray crystallographic experimental–theoretical approach is relevant to better understand the guest inclusion. It has been demonstrated for the first time that by using *kinetic control* (i.e., instant synthesis), it is possible to achieve the crystalline **M_12_L_8_** poly-[*n*]-catenane in large quantities, at a short time, and in good yields for ligand **TPP** and ZnX_2_. This is significant in the **TPP** ligand case, as slow crystallization resulted in coordination polymers when using ZnCl_2_ and ZnI_2_ but not under kinetic control. Finally, solvent-free synthesis by mechanochemical means (i.e., neat grinding) has been applied successfully for the first time to produce amorphous phases of 1D poly-[*n*]-catenanes self-assembled with **M_12_L_8_** nanocages using **TPP** and ZnX_2_ (where X = Cl, Br, and I). This has been confirmed by the exceptional dynamic behavior of the noncrystalline phases that are able to uptake and become crystalline via an *amorphous-to-crystalline* phase transformation process. The results reported herein not only provide fundamental knowledge on the structure–function relationship in **M_12_L_8_** poly-[*n*]-catenanes but also furnish two very powerful synthetic approaches that, from an industrial point of view, are quite relevant. The absence of a solvent is fundamental to move toward a *green chemistry* approach, where a toxic solvent is reduced as much as possible, as it has been shown by neat grinding.

## Experimental Section

### Single Crystal Preparation of **1·*p*-CT**

For the **1·*p*-CT** single crystal preparation, 15 mg of **TPB** was dissolved in 4 mL:1 mL of *p*-chlorotoluene:methanol. The homogeneous **TPB** solution was placed in the bottom of a crystallization tube to which a layer of methanol (3 mL) was stratified. Then, a methanolic solution of ZnBr_2_ (17 mg dissolved in 2 mL of methanol) was added dropwise. The tube was left for 5 days to stand in the lab. Optical inspection showed that single crystals were attached to the walls in the middle area of the solution where **TPB** and ZnBr_2_ were mixed after diffusion.

### Single-Crystal XRD of **1·*p*-CT**

Single-crystal X-ray data of the poly-[*n*]-catenane **1·*p*-CT** were recorded using a XtaLAB Synergy-S, Dualflex, HyPix-6000HE diffractometer. A single brown block-shaped crystal of **1·*p*-CT** was obtained by crystallization from a three-layered tube, as shown in Figure S1. A suitable crystal of 0.10 × 0.07 × 0.05 mm^3^ was selected and mounted on a suitable support on an XtaLAB Synergy-S, Dualflex, HyPix-6000HE diffractometer. The crystal was kept at steady *T* = 100.00(10) K during data collection. The structure was solved with the ShelXT^62^ structure solution program using the Intrinsic Phasing solution method and by using Olex2^63^ as the graphical interface. The model was refined with version 2014/7 of ShelXL 2014/7^62^ using least-squares minimization. Data were measured using ω scans of 0.5° per frame for 2.5/10.0 s using Cu K_α_ radiation. The total number of runs and images was based on the strategy calculation from the program CrysAlisPro.^64^ The maximum resolution that was achieved was 0.78 Å. The total number of runs and images was based on the strategy calculation from the program CrysAlisPro,^64^ and the unit cell was refined using CrysAlisPro^64^ on 18,676 reflections, 0% of the observed reflections. Data reduction, scaling, and absorption corrections were performed using CrysAlisPro.^64^ The final completeness is 99.90% out to 81.128° in Q. A Gaussian absorption correction was performed using CrysAlisPro.^64^ Numerical absorption correction was based on the Gaussian integration over a multifaceted crystal model. Empirical absorption correction using spherical harmonics was implemented in SCALE3 ABSPACK scaling algorithm. The absorption coefficient *m* of this material is 6.183 mm^–1^ at this wavelength (λ = 1.542 Å), and the minimum and maximum transmissions are 0.198 and 0.482, respectively. The structure was solved, and the space group *R*-3 (# 148) was determined by the ShelXT^62^ structure solution program using Intrinsic Phasing and refined by least squares using version 2014/7 of ShelXL 2014/7.^62^ All nonhydrogen atoms were refined anisotropically. Hydrogen atom positions were calculated geometrically and refined using the riding model. Crystal data (**1·*p*-CT**). C_196_H_148_Br_24_Cl_4_N_24_Zn_12_, *M*_r_ = 5683.46, trigonal, *R*-3 (No. 148), *a* = 37.9460(6) Å, *b* = 37.9460(6) Å, *c* = 15.7786(3) Å, α = 90°, β = 90°, γ = 120°, *V* = 19675.7(7) Å^3^, *T* = 100.00(10) K, *Z* = 3, *Z*′ = 0.166667, μ(Cu K_α_) = 6.183, 46,880 reflections measured, 9424 unique (*R*_int_ = 0.0294), which were used in all calculations. The final w*R*_2_ was 0.2298 (all data), and *R*_1_ was 0.0741 (*I* > 2(*I*)). Table S1 contains further crystallographic information. The reference CCDC code for **1·*p*-CT** is 2,097,009.

### Solution and Solid-State Synthesis of TPP-ZnX_2_ M_12_L_8_ Poly-[*n*]-catenanes

Detailed description of the instant synthesis and the solid-state preparation of the **M_12_L_8_** poly-[*n*]-catenanes using **TPP** with ZnX_2_ can be found in the Supporting Information.

### Powder X-ray Diffraction Experiments

All the powder X-ray diffraction experiments were carried out using a Bruker D2-Phaser diffractometer equipped with Cu radiation (λ = 1.54184 Å) using Bragg–Brentano geometry. The experiments were performed at room temperature.

### TG Experiments

**TG** analysis was carried out using a PerkinElmer thermal analysis instrument at the Laboratorio Analisi Chimiche at the Dipartimento di Chimica, Materiali ed. Ingegneria Chimica, Politecnico di Milano. The analyzed microcrystalline samples were heated within the temperature range from 30 to 700 °C using a heating rate of 10 °C/min under N_2_.

### Density Functional Theory

Molecular modeling studies are performed in the gas phase. The calculations rely on the gradient-corrected GGA PBE functional.^65,66^ A numerical double-zeta numerical basis set centered on atoms (including polarization functions on all atoms), roughly comparable with the usual 6-31G** Gaussian basis, has been employed. Explicit van der Waals corrections^67^ were also used to improve the description of van der Waals intraparticle interactions.^68,69^ The DMol^3^ package^70^ was employed for all the calculations.

## Supporting Information Available

Additional experimental details including photographs of **TPP M_12_L_8_** poly-[*n*]-catenane solid-state synthesis, single-crystal XRD data, powder XRD plots, and DFT-calculated MEP of a toluene guest molecule (PDF)
