# Supplementary material for: Experimental X-ray and DFT Structural Analyses of M12L8 Poly-[n]-catenanes Using exo-Tridentate Ligands
Source: Inorg Chem. 2022 Jun 30;61(28):10863–71. doi: 10.1021/acs.inorgchem.2c01290 (PMC9937537; doi:10.1021/acs.inorgchem.2c01290)
Supplement: Supplementary file 1 — ic2c01290_si_001.pdf [file ic2c01290_si_001.pdf]

Supporting Information

"Experimental X-ray and DFT structural analyses of $M_{12}L_8$ poly-[*n*]-catenanes using exo-tridentate ligands"

Javier Martí-Rujas*[†,□],Sijie Ma,[†] Antonino Famulari*[†,#]

† Dipartimento di Chimica Materiali e Ingegneria Chimica ''Giulio Natta'', Politecnico di Milano, Via L. Mancinelli 7, 20131 Milan, Italy.

□ Center for Nano Science and Technology@Polimi, Istituto Italiano di Tecnologia, Via Pascoli 70/3, 20133 Milano, Italy.

INSTM Consorzio Interuniversitario Nazionale per la Scienza e Tecnologia dei Materiali

*E-mail: javier.marti@polimi.it; antonino.famulari@polimi.it

Contents

Synthesis of TPB-$ZnBr_2$ poly-[*n*]-catenane in the solution state (layering crystallization method).

Single crystal XRD description of poly-[*n*]-catenane 1·*p*-CT.

Single crystal preparation of poly-[*n*]-catenane 1·Tol.

Single crystal XRD description of poly-[*n*]-catenane 1·Tol.

Instant synthesis of $M_{12}L_8$ poly-[n]-catenanes using TPP ligand with ZnX_2 (where X = Cl, Br, I).

Instant synthesis using 1,2-dichlorobenzene as templating molecule.

Mechanochemical synthesis using TPP ligand and ZnX_2 (where X = I, Br and Cl) in absence of solvent (neat grinding).

Table S1. Crystallographic data of **1·*p*-CT**.

References

List of Figures

Figure S1. (Top) Experimental powder XRD pattern of ligand 2,4,6-tris-(4-pyridyl)pyridine (**TPP**) measured at room temperature. The sample is the one purchased from iChemicals. The experimental powder XRD pattern fits well with the simulated from the crystal structure of **TPP** (100 K) reported by Zaworotko and coworkers (CCDC Code: UBEJUK) depicted at the bottom.

Figure S2. Crystallization set up of **1·*p*-CT**. The aromatic solvent used for the templating effect is *p*-chlorotoulene. The observed crystals showed a block-shape, sometimes observed as icosahedral prisms.

Figure S3. Simulated powder XRD pattern of **1·*p*-CT** (100 K).

Figure S4. (Top) Crystal structure of **1·*p*-CT** showing the packing of the 1D chanis of interlocked $M_{12}L_8$ nanocages viewed along the *c*-axis. The voids in the central cages

corresponding to the disordered guest molecules not resolved by SC-XRD are showed in yellow. The void in the $\mathbf{M_{12}L_8}$ nanocage is *ca*. 17.2 % of the total unit cell volume. (Bottom) Voids in **1·*p*-CT** after manually removing the guests molecules. The volume occupied by the *p*-chlorotoluene guests is *ca*. 33.8 % of the total unit cell volume. Notice, like in the other reported isostructural $\mathbf{M_{12}L_8}$ **TPB** polycatenanes that the voids are not connected among each other.

Figure S5. Crystal structure of **1·*p*-CT** showing 6 *p*-chlorotoluene guest molecules with occupancy factor of 0.6667 included in one $\mathbf{M_{12}L_8}$ nanocage viewed along the *c*-axis (a) and along *a*-axis (b).

Figure S6. a) Crystal structure of **1·*p*-CT** showing the good face-to-face orientation of the central benzene rings in the **TPB** ligand among two $\mathbf{M_{12}L_8}$ interlocked nanocages. The carbon atoms belonging to different cages are depicted in green and orange. b) Disorder observed in the ligand **TPB** and $ZnBr_2$ in the $\mathbf{M_{12}L_8}$ nanocages.

Figure S7. Single crystal X-ray structure of **1·Tol** recorded at room temperature**.** a) Asymmetric unit and b) $\mathbf{M_{12}L_8}$ nanocage containing six toluene guest molecules. It is important to note that the toluene guest molecule is oriented as expected according the computed MEPs for the ***p*-CT** and ***p*-CA** guests containing a methyl group (see Figure S7).

Figure S8. DFT calculated MEP of toluene guest molecule. The electropositive and electronegative regions are represented in blue and in red respectively.

Figure S9. a) Plot depicting the lattice parameters for the *c*-axis and b) plots corresponding to *a* and *b*-axis for the 100 K temperature structures **1·*p*-CT, 1·*o*-DCB, 1·*p*-CA** and the room temperature data structure including toluene **1·Tol.**

Figure S10. Powder XRD pattern obtained upon instant synthesis using **TPP**, $ZnBr_2$ with *o*-DCB/MeOH. The powder XRD was measured at room temperature.

Figure S11. Powder XRD pattern obtained upon instant synthesis using **TPP**, $ZnCl_2$ with *o*-DCB /MeOH. The powder XRD was measured at room temperature.

Figure S12. Powder XRD pattern obtained upon instant synthesis using **TPP**, ZnI_2 with *o*-DCB /MeOH. The powder XRD was measured at room temperature.

Figure S13. (a) Experimental powder XRD obtained upon instant synthesis of **TPP** and $ZnBr_2$ using *o*-DCB as templating molecule (**2·o-DCB**). (b) Experimental powder XRD pattern of the **TPB**-$ZnBr_2$ poly-[*n*]-catenane including *o*-DCB as templating molecule. The powder XRD were measured at room temperature.

Figure S14. Pictures taken during the grinding of **TPP** and ZnI_2. The grinding process gives a powder product that can be manipulated very well (*i.e.*, transferred from the mortar to the vial).

Figure S15. Pictures taken during the grinding of **TPP** and $ZnBr_2$. In this case the grinding process gives a powder product that can be manipulated well (*i.e.*, transferred from the mortar to the vial).

Figure S16. Pictures taken during the grinding of **TPP** and $ZnCl_2$. This can be seen from the final weight. The grinding process gives a powder product that can be manipulated very well (*i.e.*, transferred from the mortar to the vial).

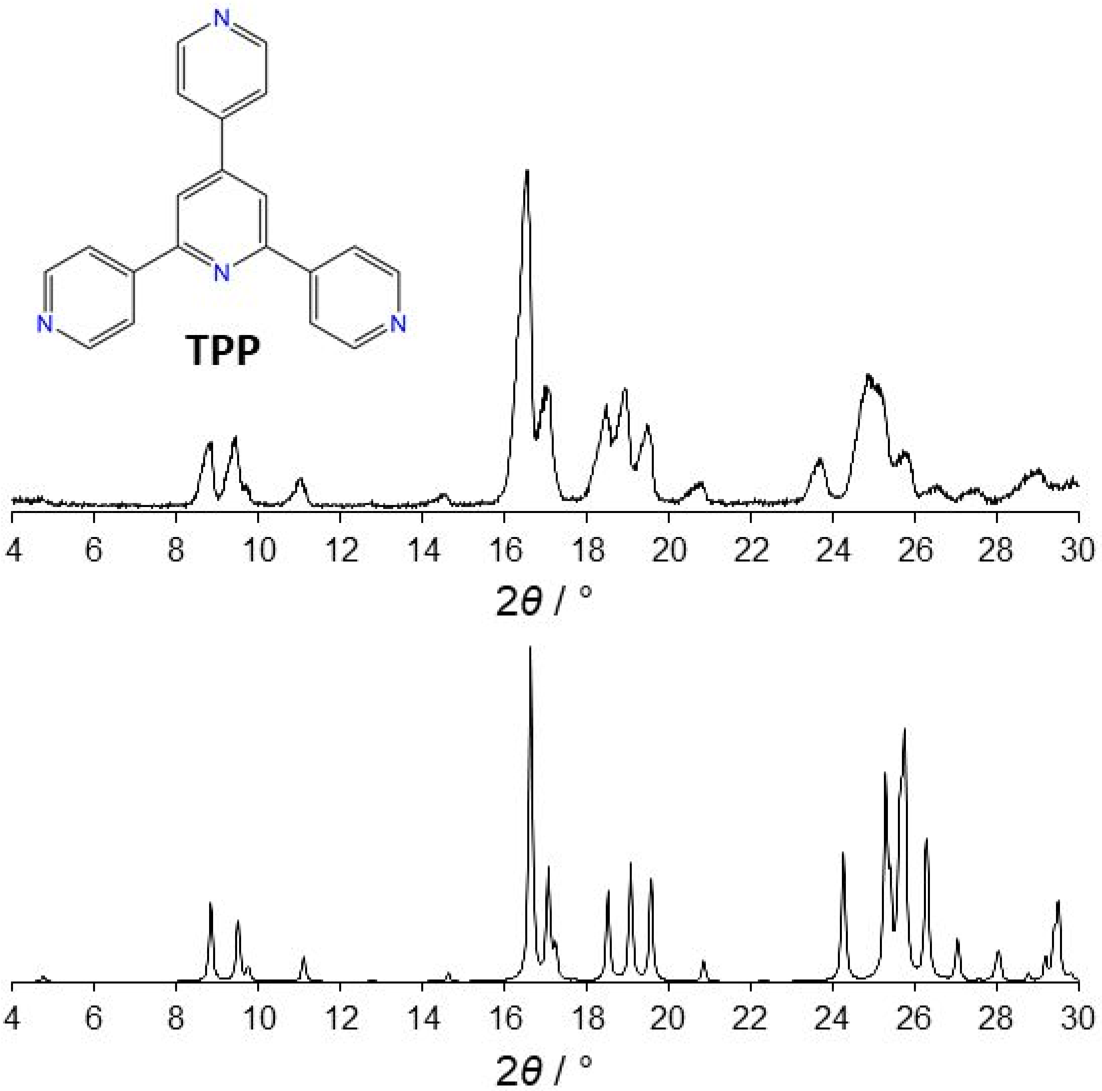

Figure S1. (Top) Experimental powder XRD pattern of ligand 2,4,6-tris-(4-pyridyl)pyridine (**TPP**) measured at room temperature. The sample is the one purchased from iChemicals. The experimental powder XRD pattern fits well with the simulated from the crystal structure of **TPP** (100 K) reported by Zaworotko and coworkers (CCDC Code: UBEJUK) depicted at the bottom.[1]

Synthesis of TPB-$ZnBr_2$ poly-[*n*]-catenane in the solution state (layering crystallization method).

Poly-[*n*]-catenane (1·*p*-CT) single crystal preparation.

For the **1·*p*-CT** single crystal preparation, 15 mg of **TPB** were dissolved in 4 ml :1 ml of *p*-chlorotoluene:methanol. The homogenous **TPB** solution was placed in the bottom of a crystallization tube to which a layer of methanol (3 ml) was stratified. Then a methanolic solution of $ZnBr_2$ (17 mg dissolved in 2 ml

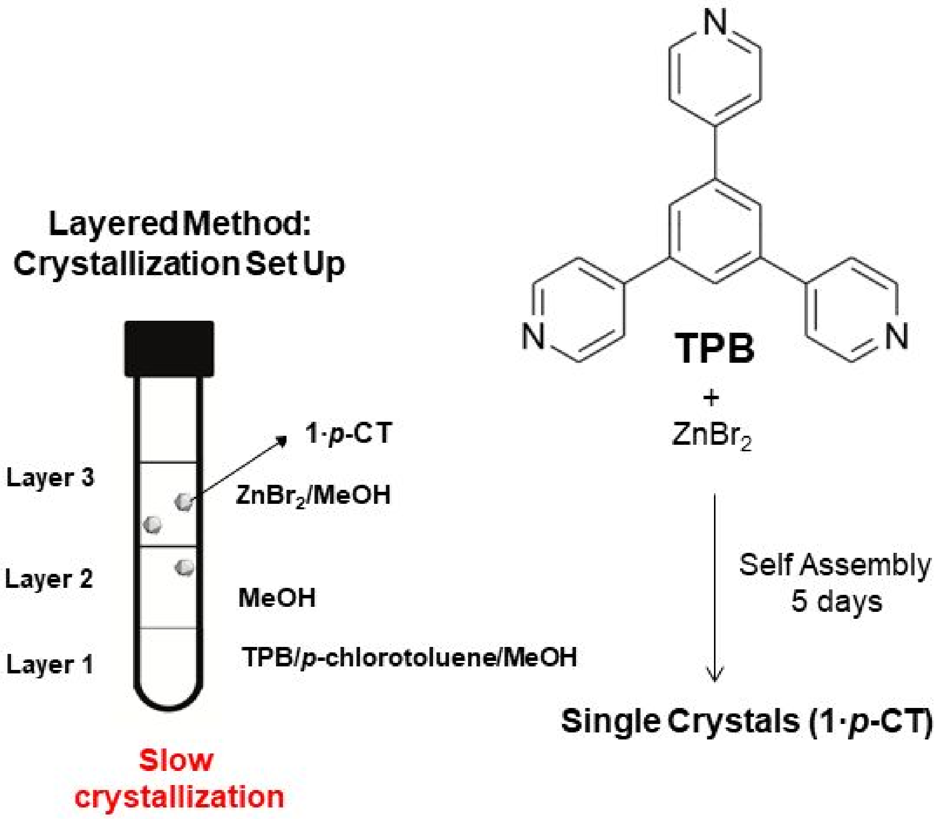

Figure S2. Crystallization set up of **1·*p*-CT**. The aromatic solvent used for the templating effect is *p*-chlorotoulene. The observed crystals showed a block-shape, sometimes observed as icosahedral prisms.

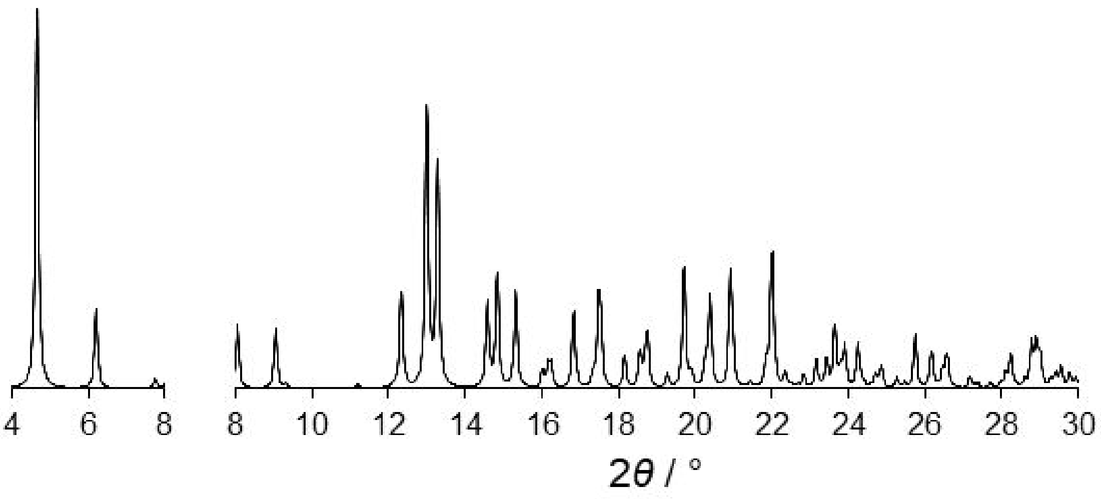

Figure S3. Simulated powder XRD pattern of **1·*p*-CT** (100 K)**.**

Single crystal XRD description of poly-[*n*]-catenane 1·*p*-CT.

The crystal structure of **1·*p*-CT** is isostructural to the **TPB**-$ZnCl_2$ including nitrobenzene reported recently[2]. The open windows considering the distance among Zn atoms have large apertures of 13.474 Å × 21.541 Å. In a $\mathbf{M_{12}L_8}$ single cage (*i.e.*, non-interlocked), the distance among the two benzene rings in the **TPB** ligands is 20.099 Å.

The nanocages are doubly-interlocked and expand along the [001] crystallographic direction. Removing in silico the guest molecules from the $\mathbf{M_{12}L_8}$ nanocages the *free* volume obtained is 6656.52 Å^3 (*i.e.*, 33.8 % of unit cell volume) as shown in Figure S2. Importantly, the 100 K structure of **1·*p*-CT** does not have continuous channels.

One *p*-chlorotoluene guest molecule in the asymmetric unit is ordered and can be resolved by X-ray crystallography (100 K). The *p*-chlorotoluene occupancy refine to around 0.69 – 0.71 values.

As shown in Figure S3, in the **$M_{12}L_8$** nanocage the ordered solvent molecules are arranged in a hexagonal symmetry, whereas the top and bottom parts of the nanocage are free of solvent because it is the area where the nanocages are concatenated. The six included guest molecules are “glued” via aromatic-aromatic interactions while the two left **TPB** ligands are not interacting with *p*-chlorotoluene guests because they are involved in the interlocking of the **$M_{12}L_8$** cages. For this reason, the shape of the **$M_{12}L_8$** voids is like a flattened sphere. The disordered *p*-chlorotoluene molecules not possible to resolve by X-ray data are located in the central part of the cages. The templating effect of large aromatic *p*-chlorotoluene is crucial for maintaining the large voids stable, even at room temperature.

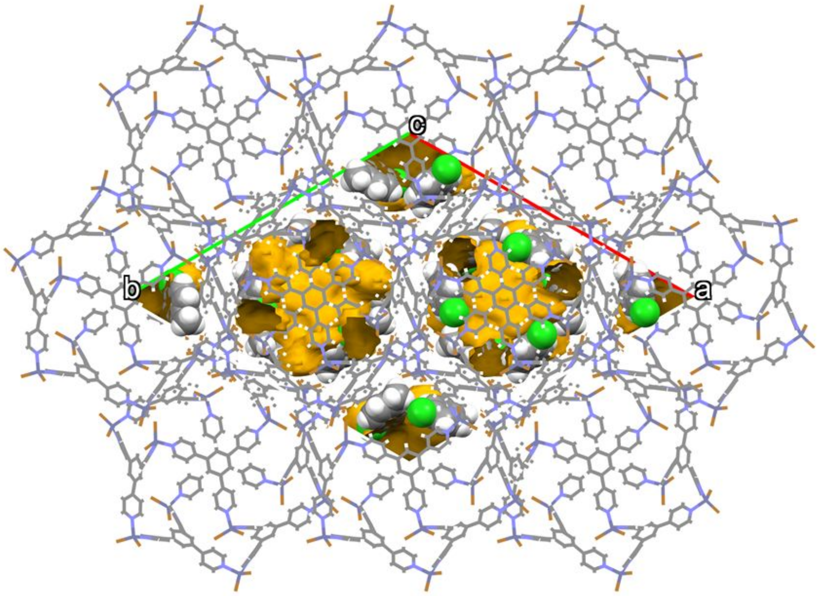

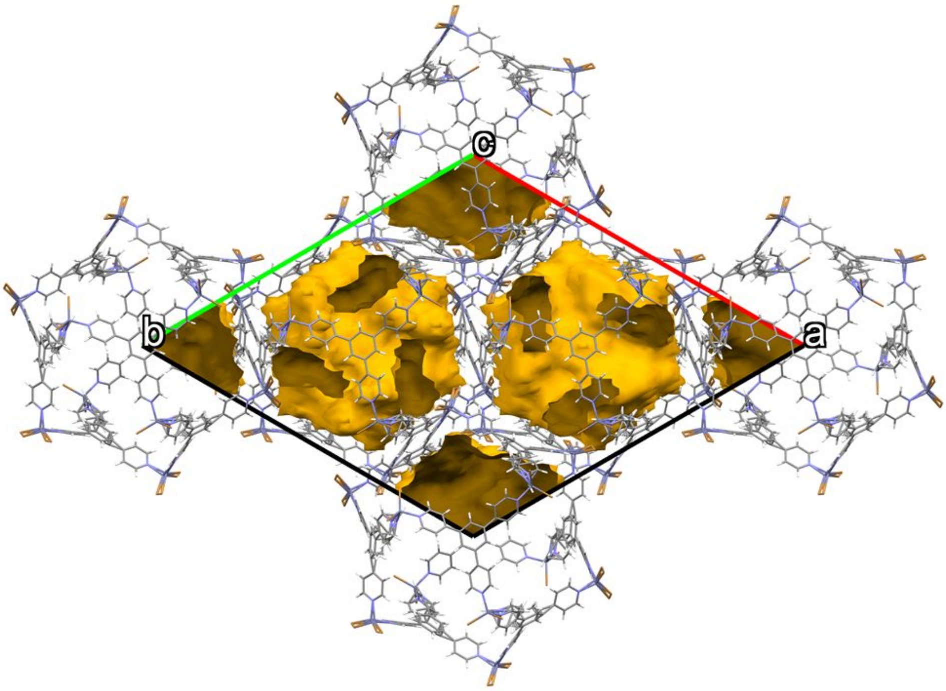

Figure S4. (Top) Crystal structure of **1·*p*-CT** showing the packing of the 1D chanis of interlocked $\mathbf{M_{12}L_8}$ nanocages viewed along the *c*-axis. The voids in the central cages corresponding to the disordered guest molecules not resolved by SC-XRD are showed in yellow. The void in the $\mathbf{M_{12}L_8}$ nanocage is *ca*. 17.2 % of the total unit cell volume. (Bottom) Voids in **1·*p*-CT** after manually removing the guests molecules. The volume occupied by the *p*-chlorotoluene guests is *ca*. 33.8 % of the total unit cell volume. Notice, like in the other reported isostructural $\mathbf{M_{12}L_8}$ **TPB** polycatenanes that the voids are not connected among each other.

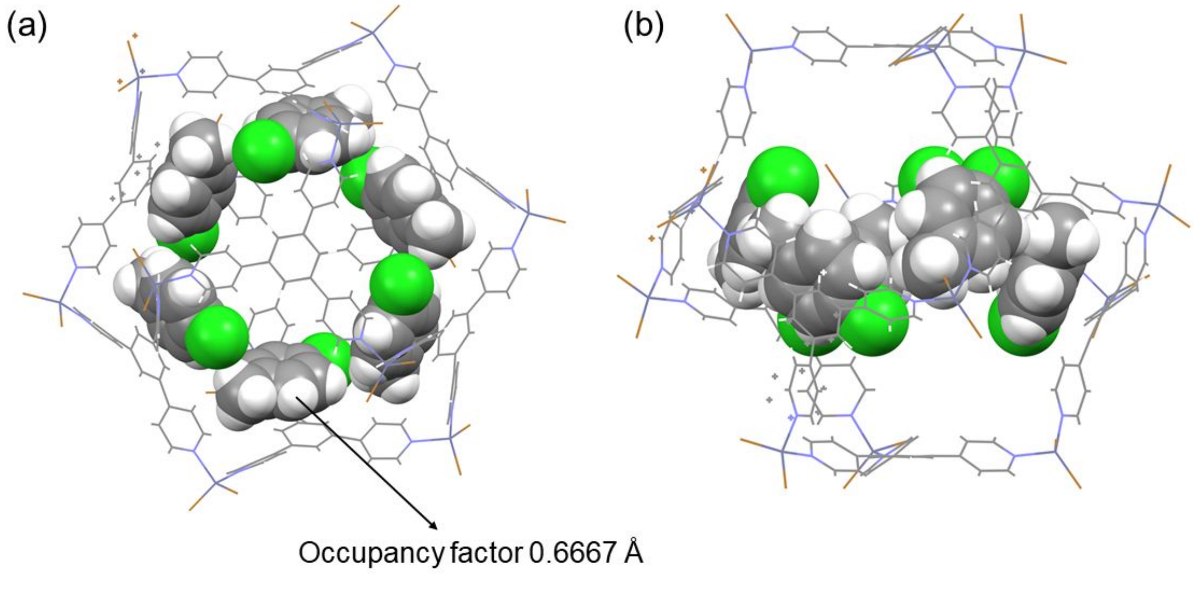

Figure S5. Crystal structure of **1·*p*-CT** showing 6 *p*-chlorotoluene guest molecules with occupancy factor of 0.6667 included in one $\mathbf{M_{12}L_8}$ nanocage viewed along the *c*-axis (a) and along *a*-axis (b).

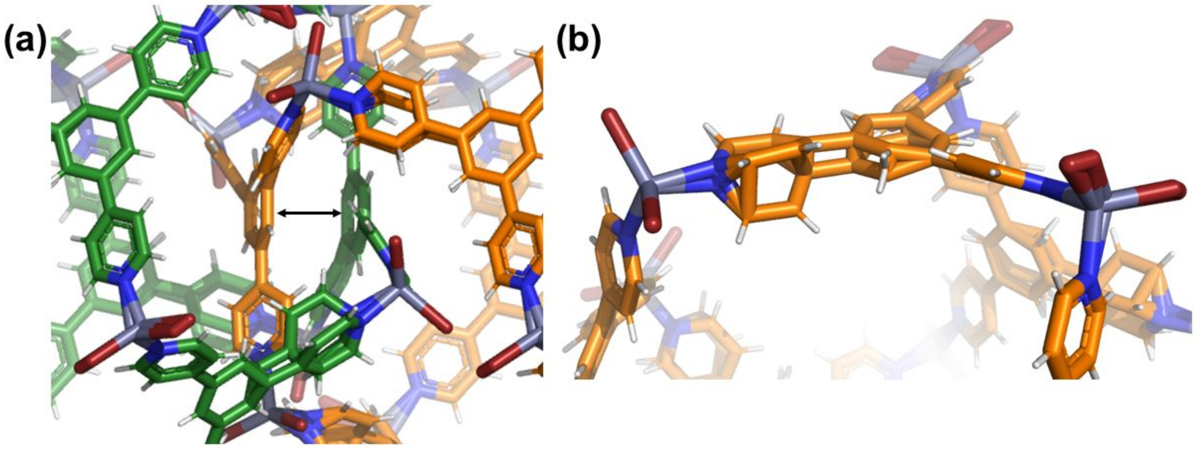

TPB benzene-benzene
Aromatic distance: 3.839 Å

Disordered pyridine-benzene TPB ring and $ZnBr_2$ in $M_{12}L_8$ nanocages.

Figure S6. a) Crystal structure of **1·*p*-CT** showing the good face-to-face orientation of the central benzene rings in the **TPB** ligand among two $\mathbf{M_{12}L_8}$ interlocked nanocages. The carbon atoms belonging to different cages are depicted in green and orange. b) Disorder observed in the ligand **TPB** and $ZnBr_2$ in the $\mathbf{M_{12}L_8}$ nanocages.

Single crystal preparation of poly-[*n*]-catenane 1·Tol.

For the **1·Tol** single crystal preparation, 15 mg of **TPB** were dissolved in 5 ml :1 ml of toluene:methanol. The homogenous **TPB** solution was placed in the bottom of a crystallization tube to which a layer of methanol (3 ml) was stratified. Then a methanolic solution of $ZnBr_2$ (17 mg dissolved in 2 ml of methanol) was added dropwise. The tube was left for 5 days to stand in the lab. Optical inspection showed large single crystals attached to the walls in the middle area of the solution where the **TPB** and $ZnBr_2$ were mixed after diffusing**.**

Single crystal XRD description of poly-[*n*]-catenane 1·Tol.

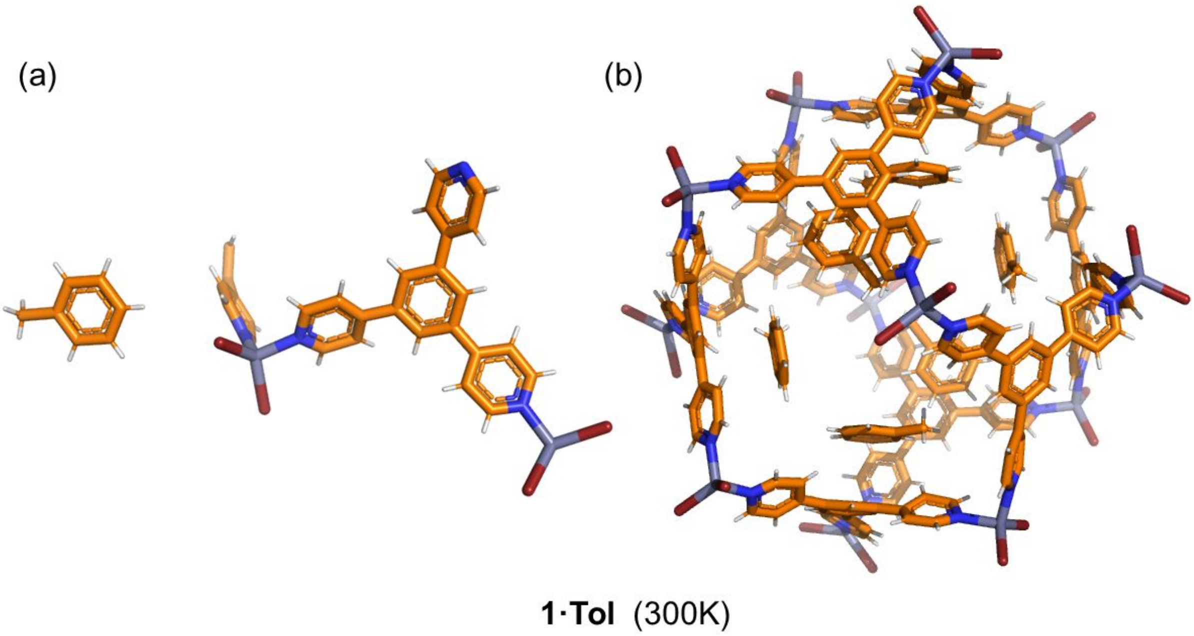

1·Tol (300K)

Figure S7. Single crystal X-ray structure of **1·Tol** recorded at room temperature**.** a) Asymmetric unit and b) $\mathbf{M_{12}L_8}$ nanocage containing six toluene guest molecules. It is important to note that the toluene guest molecule is oriented as expected according the computed MEPs for the ***p*-CT** and ***p*-CA** guests containing a methyl group (see Figure S7).

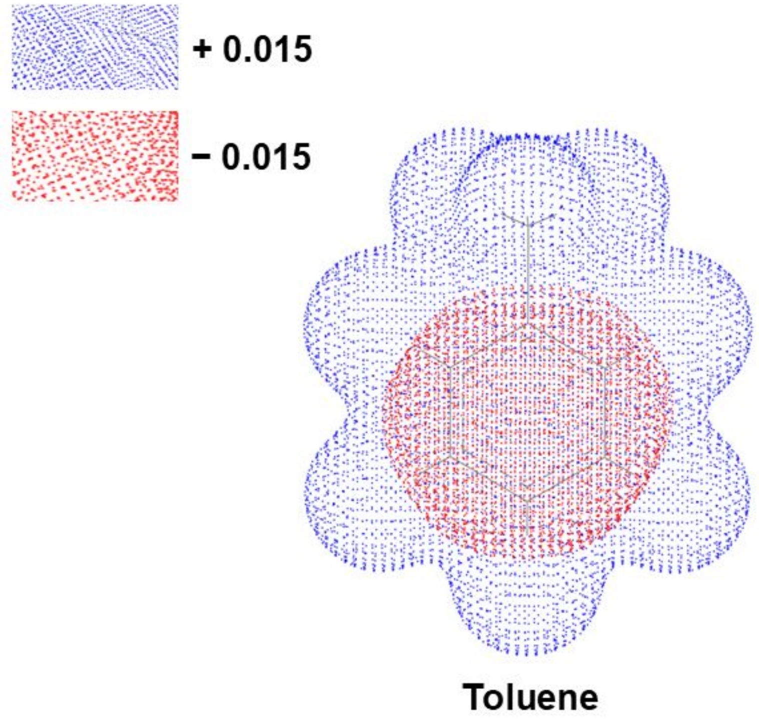

Figure S8. DFT calculated MEP of toluene guest molecule. The electropositive and electronegative regions are represented in blue and in red respectively.

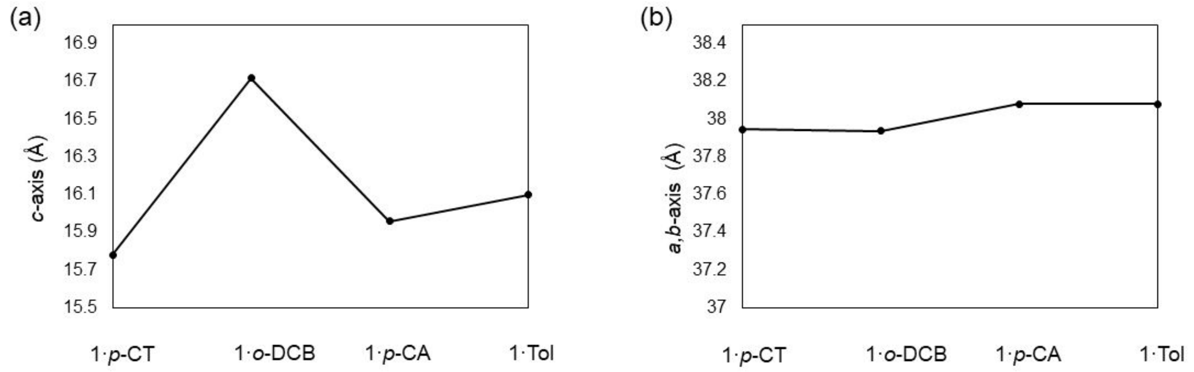

Figure S9. a) Plot depicting the lattice parameters for the *c*-axis and b) plots corresponding to *a* and *b*-axis for the 100 K temperature structures **1·*p*-CT, 1·*o*-DCB, 1·*p*-CA** and the room temperature data structure including toluene **1·Tol.**

Instant synthesis of $M_{12}L_8$ poly-[n]-catenanes using TPP ligand with ZnX_2 (where X = Cl, Br, I).

Instant synthesis using 1,2-dichlorobenzene as templating molecule.

Synthesis the **TPP**-$ZnBr_2$ poly-[*n*]-catenane: 30 mg of **TPP** were mixed in 10 ml of 1,2-dichlorobenzene and 4 ml of methanol and stirred at room temperature for 5 minutes until the suspension became clear. Then to the stirring **TPP** solution a methanolic solution of $ZnBr_2$ (33 mg in 2 ml) was added instantaneously all at once. Immediately a precipitate was formed. After filtration the white sample was analyzed by powder XRD data as shown in Figure S9. The yield based on **TPP** ligand is (55 mg; 60 %).

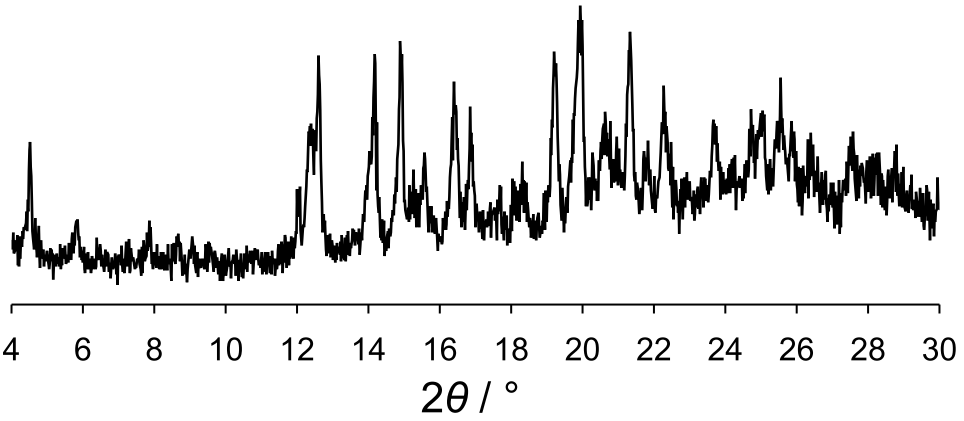

Figure S10. Powder XRD pattern obtained upon instant synthesis using **TPP**, $ZnBr_2$ with *o*-DCB/MeOH. The powder XRD was measured at room temperature.

Synthesis the **TPP**-$ZnCl_2$ poly-[*n*]-catenane: 30 mg of **TPP** were mixed in 10 ml of 1,2-dichlorobenzene and 4 ml of methanol and stirred at room temperature for 5 minutes until the suspension became homogeneous. Then to the vigorous stirred **TPP** solution a methanolic solution of $ZnCl_2$ (20 mg in 2 ml) was added instantaneously. Immediately the solution turned a suspension after a precipitate was formed. Following the filtration, the white sample was analyzed by powder XRD data as shown in Figure S10. The yield based on **TPP** ligand is (49 mg; 62 %).

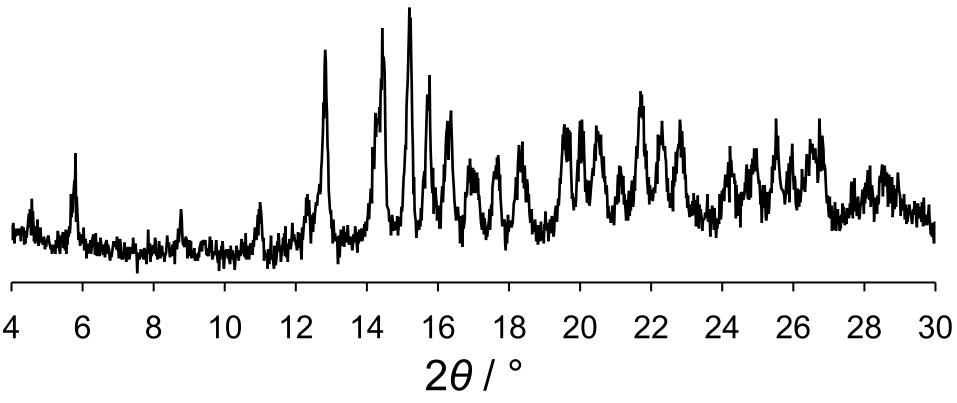

Figure S11. Powder XRD pattern obtained upon instant synthesis using **TPP**, $ZnCl_2$ with *o*-DCB /MeOH. The powder XRD was measured at room temperature.

<u>Synthesis the **TPP**-ZnI_2 poly-[*n*]-catenane</u>: 30 mg of **TPP** were mixed in 10 ml of 1,2-dichlorobenzene and 4 ml of methanol and stirred at room temperature for 5 minutes until the suspension became homogeneous. Then to the vigorous stirred **TPP** solution a methanolic solution of ZnI_2 (47 mg in 2 ml) was added instantaneously. Immediately a precipitate was formed. After filtration the white sample was analyzed by powder XRD data as shown in Figure S11. The yield based on **TPP** ligand is (68 mg; 64 %).

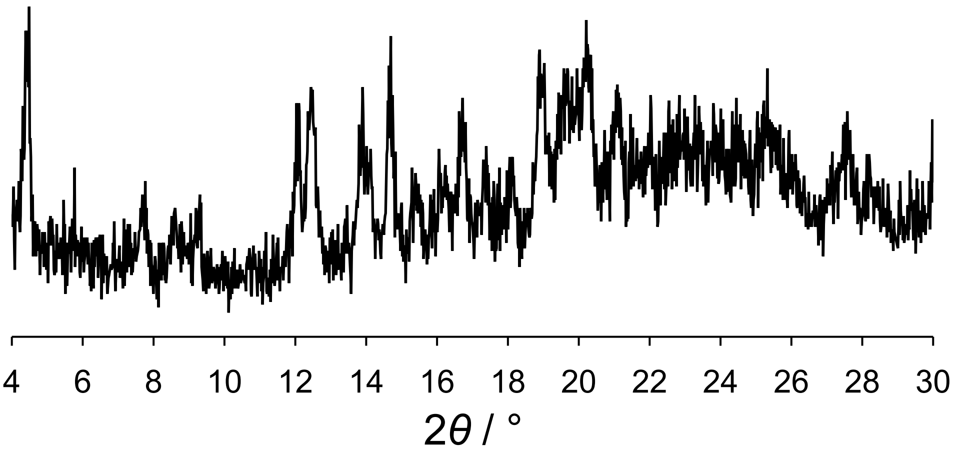

Figure S12. Powder XRD pattern obtained upon instant synthesis using **TPP**, ZnI_2 with *o*-DCB /MeOH. The powder XRD was measured at room temperature.

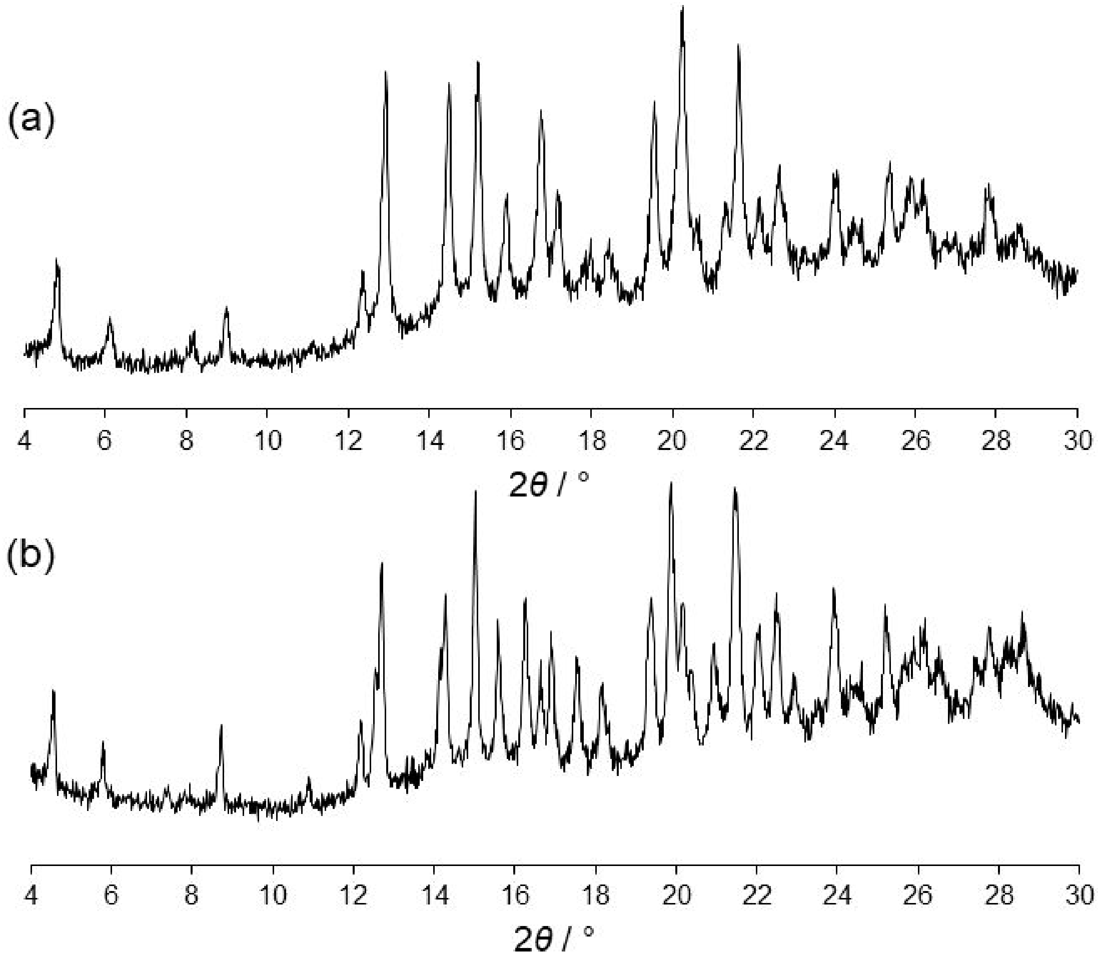

Figure S13. (a) Experimental powder XRD obtained upon instant synthesis of **TPP** and $ZnBr_2$ using *o*-DCB as templating molecule (**2·*o*-DCB**). (b) Experimental powder XRD pattern of the **TPB**-$ZnBr_2$ poly-[*n*]-catenane including *o*-DCB as templating molecule. The powder XRD were measured at room temperature.

Mechanochemical synthesis using TPP ligand and ZnX_2 (where X = I, Br and Cl) in absence of solvent (neat grinding).

Neat grinding of TPP and ZnI_2: For the neat grinding synthesis, 30 mg of **TPP** were ground with ZnI_2 (47 mg) for 15 minutes using a mortar and pestle (Figure S13). During the grinding, the samples were mixed with a spatula to achieve the best reactivity among reactants as after *ca*. 1 minute of grinding the solid remains attached to the surface of the mortar. The color of the product is light yellow (Figure S13). Obtained weight after grinding: 76 mg; (Yield 99 %). The sample was put in a filter paper in a funnel and further washed with a mixture of methanol (4ml) and chloroform (4ml) and left to equilibrate with atmosphere for 1 day. The weight after washing with methanol and chloroform is 54 mg (Yield 70 %).

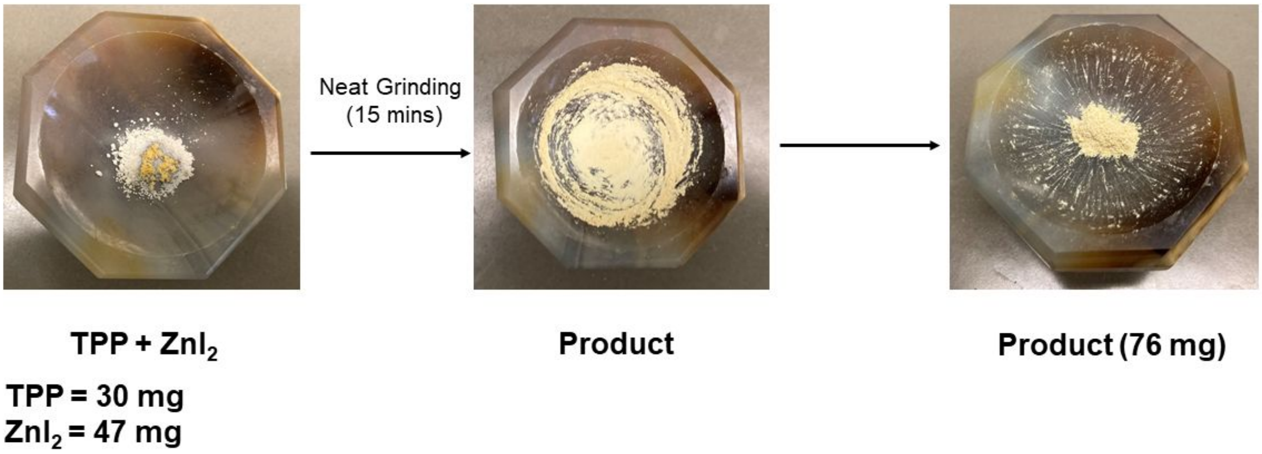

Figure S14. Pictures taken during the grinding of **TPP** and ZnI_2. The grinding process gives a powder product that can be manipulated very well (*i.e.*, transferred from the mortar to the vial).

Neat grinding of TPP and $ZnBr_2$: For the neat grinding reaction, 30 mg of **TPP** were ground with $ZnBr_2$ (32.767 mg) for 15 minutes using a mortar and pestle. As in the previous case, during the grinding the samples were mixed homogeneously with a spatula to achieve the best reactivity among reactants as after *ca*. 1 minute of grinding the solid remains attached to the surface of the mortar. The color of the product is white (Figure S14). Obtained weight after grinding: 58 mg (Yield: 92.25 %). The sample was put in a filter paper in a funnel and further washed with a mixture of methanol (4ml) and chloroform (4ml) and left to equilibrate

with atmosphere for 1 day. The weight after washing with methanol and chloroform is 46 mg (Yield 73 %).

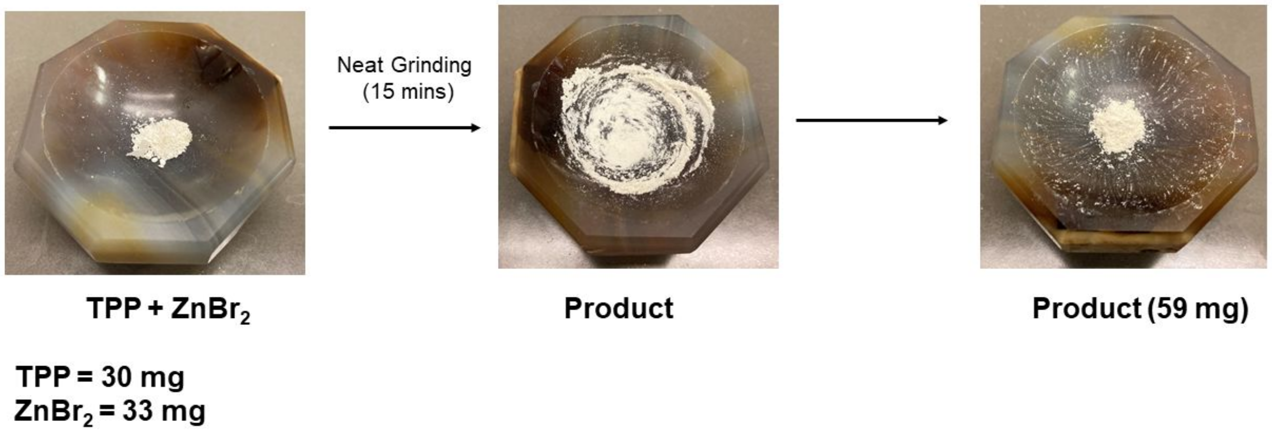

Figure S15. Pictures taken during the grinding of **TPP** and $ZnBr_2$. In this case the grinding process gives a powder product that can be manipulated well (*i.e.*, transferred from the mortar to the vial).

Neat grinding of TPP and $ZnCl_2$: For the neat grinding synthesis, 30 mg of **TPP** were ground with $ZnCl_2$ (20 mg) for 15 minutes using a mortar and pestle. The grinding procedure was as in the $ZnBr_2$ and ZnI_2 cases described above. The product is white (Figure S15). Obtained weight after grinding: 49 mg (Yield: 98.14 %). The weight after washing with methanol and chloroform is 35 mg (Yield 72.1 %).

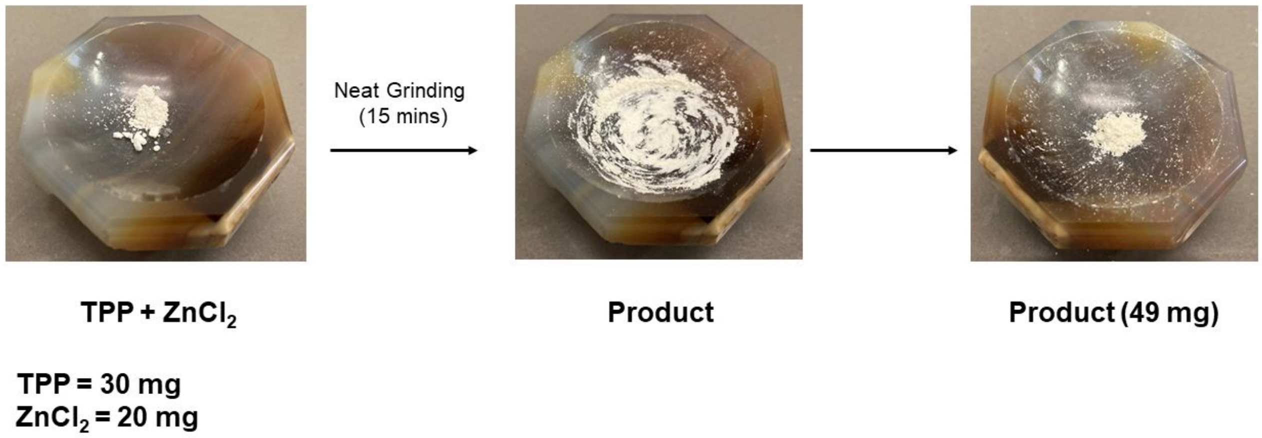

Figure S16. Pictures taken during the grinding of **TPP** and $ZnCl_2$. This can be seen from the final weight. The grinding process gives a powder product that can be manipulated very well (*i.e.*, transferred from the mortar to the vial).

Note about the neat grinding reaction: Our estimation of product that cannot be recovered after collecting the mechanochemical product and after washing it is *ca*. 2-3 mg, which means that the actual yields are slightly higher.

Table S1. Crystallographic data of **1·*p*-CT**.

**Compound**	**1·*p*-CT**
Formula	$C_{196}H_{148}Br_{24}Cl_4$ $N_{24}Zn_{12}$
$D_{calc.}$/ g cm^{-3}	1.439
m/mm^{-1}	6.183
Formula Weight	5683.46
Colour	brown
Shape	block
Size/mm^3	0.10×0.07×0.05
T/K	100.00(10)
Crystal System	trigonal
Space Group	*R*-3
a/Å	37.9460(6)
b/Å	37.9460(6)
c/Å	15.7786(3)
a/°	90
b/°	90
g/°	120
V/Å^3	19675.7(7)
Z	3
Z'	0.166667
Wavelength/Å	1.54184
Radiation type	Cu K_{α}
Q_{min}/°	3.884
Q_{max}/°	81.128
Measured Refl.	46880
Independent Refl.	9424
Reflections with I > 2(I)	7302
R_{int}	0.0294
Parameters	499
Restraints	126
Largest Peak	0.686
Deepest Hole	-0.925
GooF	1.061
wR_2 (all data)	0.2298
wR_2	0.2211
R_1 (all data)	0.0847
R_1	0.0741

References

1 Sanii, R.; Patyk-Kazmierczak, E.; Hua, C.; Darwish, S.; Pham, T.; Forrest, K. A.; Space, B.; Zaworotko, M.J. Toward an Understanding of the Propensity for Crystalline Hydrate Formation by Molecular Compounds. Part 2. *Cryst.Growth Des*. **2021**, *21*, 4927-4939.

2 Torresi, S.; Famulari, A.; Martí-Rujas, J. Kinetically controlled fast crystallization of $M_{12}L_8$ poly-[*n*]-catenanes using the 2,4,6-tris(4-pyridyl)benzene ligand and $ZnCl_2$ in an aromatic environment. *J. Am. Chem. Soc.*, **2020**, *142*, 9537-9543.
